# A Doctor's Fear of Premature Cremation

**Published:** 1915-05-15

**Authors:** Jas. R. Williamson

**Affiliations:** 100 Chedington Road, Upper Edmonton, London, N.


*To the Editor of* The Hospital.

### "A Doctor's Fear of Premature Cremation."

*To the Editor of* The Hospital.

Sir,—With reference to the interesting report under the above title in your instructive journal last week, if one may judge by the extent of correspondence and comments in the Press and the numerous inquiries for information, the question of reform in the burial laws, so as to ensure effective verification of death in all cases, appears to have taken a firm hold on the public mind. Nearly every day, and for years past, letters reach the present writer from persons who are anxiously concerned in the movement for the prevention of premature burial. A majority emanate from women, who, according to medical observation and authority, are more liable to conditions of trance, death-like exhaustion, syncope, and other forms of suspended animation, which in some instances appear to be a suspension of life, as none of the ordinary so-called signs of death seem to apply. Not a few correspondents relate cases of narrow escape from interment alive in their own families, and it is usually through witnessing the liability to such terrible occurrences that the writers have first had their attention called to the subject. Clearly this reform, which has now been for many years demanded in the interests of humanity and for the safety of the public, should be made a matter of urgency by Parliament.—I am, Sir, your obedient servant, Jas. R. Williamson.

100 Chedington Road, Upper Edmonton,
London, N., May 10, 1915.

